# COVID-19 Pandemic and Its Gendered Impact on Indian Physicians

**DOI:** 10.1200/GO.21.00020

**Published:** 2021-07-06

**Authors:** Sabita Jiwnani, Priya Ranganathan, Virendra Tiwari, Apurva Ashok, Devayani Niyogi, George Karimundackal, C. S. Pramesh

**Affiliations:** ^1^ Division of Thoracic Surgery, Department of Surgical Oncology, Tata Memorial Centre, Homi Bhabha National Institute, Mumbai, India; ^2^ Department of Anaesthesiology, Critical Care and Pain, Tata Memorial Centre, Homi Bhabha National Institute, Mumbai, India

## Abstract

The 2018 WHO health workforce report analyzing gender equity in 104 countries reported that although women constituted 70% of the workers, they were less likely to be employed full-time and faced a 28% gender pay gap. The ongoing COVID-19 pandemic has affected professional as well as personal lives of physicians. We conducted a survey among Indian physicians to understand this impact.

**METHODS:**

A 31-point anonymized survey to evaluate the impact of the COVID-19 pandemic and resultant lockdown on physicians' domestic responsibilities was disseminated via e-mail and text messaging applications. Our aim was to evaluate whether the impact was gender-based and to look for differences in aspects of domestic work, childcare, and professional commitments.

**RESULTS:**

We obtained 1,041 responses, of which 643 identified themselves as men and 393 as women. An increase in the domestic responsibilities during the lockdown was confirmed by 90% of the women compared with 82% men. More women than men were solely responsible for domestic chores (38.7% *v* 23.7%), managed their children's education (74% *v* 31%), and felt an adverse impact of the pandemic on their professional work (60.8% *v* 42.6%). Fewer women's spouses (57/359) than men's (174/594, *P* = .00001) were forced to take leave or work reduced hours, and double the proportion of women (3.5% *v* 1.5%) had to quit their jobs to manage responsibilities at home.

**CONCLUSION:**

As the COVID-19 pandemic and the lockdown measures threw newer challenges, more women physicians than men (81% *v* 63%) shouldered the burden of increased domestic work and childcare. This survey highlights the need to re-examine the specific challenges faced by women physicians and identify means to support and empower them.

## INTRODUCTION

India witnessed a sudden and complete lockdown to combat the COVID-19 pandemic in late March 2020. Although this unplanned shutdown affected the entire country as a whole, physicians, as a group, were faced with a dual challenge: to continue to provide medical care and to also manage the inevitable increase in domestic responsibilities.^1^

**CONTEXT**

**Key Objective**
The COVID-19 pandemic adversely affected professional as well as personal lives of physicians across the world. A nationwide lockdown prevented Indian physicians to have access to domestic help as well as schooling and daycare facilities for their children. We designed this survey to evaluate the increase in domestic responsibilities and examine whether there were any gender-based differences among physicians.
**Knowledge Generated**
Our survey of Indian physicians demonstrated that disproportionately larger percentage of women physicians compared with men were responsible for the increased domestic work as well as childcare, including children's educational activities. More women than men felt an adverse impact on their work, were forced to take leave, and had to quit their jobs.
**Relevance**
The pandemic has deepened the disparity chasm among men and women physicians, with repercussions on maintaining work-life balance as well as negating the advancements made toward gender equity in medicine.

Women around the world, especially mothers, regardless of employment status, have traditionally been the primary caregivers for their families. They are expected to multitask and cope with household chores, childcare, and education, often alone and with little or no support.^2,3^ Most urban households in India, especially those with working women, rely on extended family and domestic help, either part-time or full-time, for housework related to childcare, cleaning, and cooking.^4^

Several studies suggest that women in medicine shoulder more of the burden of housework than men. A National Institutes of Health–funded physician-researchers study showed that women spent 8.5 hours more, per week, on parenting and domestic tasks than their men peers.^5^ Recent research also suggests that women take on more domestic responsibilities than men, even in dual-career academic families.^6^ Therefore, the current restrictions in access to childcare might reasonably be expected to have disproportionate impact on women in medicine, as compared to men.^7^ Studies conducted in the United States and Europe have shown that the pandemic is expected to widen the gender disparity and affect women more adversely than men, even in highly educated cohorts such as physicians.^8,9^ There were more than 1 million registered medical practitioners (MBBS) registered with the Medical Council of India as of March 31, 2017,^10^ with 27.7% of them being women.^11^

There are no data on the impact of the pandemic and resultant lockdown on the domestic burden shouldered by women physicians in India.

## METHODS

We designed a questionnaire to study the impact of the pandemic and the subsequent lockdown on domestic responsibilities of physicians, to evaluate whether there are gender-based differences and to understand the difficulties and challenges faced by physicians during this time.

The 31-point questionnaire (Data Supplement) consisted of questions pertaining to the demographics, family setup, availability of domestic help, management of domestic chores, childcare, and children's education, both prelockdown and postlockdown. We also had questions regarding the impact of the pandemic and resultant increase in domestic responsibilities on their professional duties. The study was approved by the institutional ethics committee.

The survey was shared via the following formats

1. e-mailed to a database consisting of the physicians who had previously attended oncology meetings organized by our institution, a tertiary care cancer center. Of the 8,200 e-mails sent, 3,300 bounced back or could not be delivered.

2. The survey was shared on WhatsApp (WhatsApp LLC, Facebook, Inc, Menlo Park, CA) on multiple physician groups and then forwarded by the individual physicians to other groups.

Participation in the survey was voluntary and completing the questionnaire implied consent. The survey was anonymously submitted online with no identifiers. The link needed the individual to access the survey on Google docs, where e-mail addresses of respondents were not collected as prespecified. The questionnaire was initially distributed in the third week of October 2020 with a reminder e-mail sent 1 week later; responses were collected for a period of 2 weeks from the first e-mail or message. The responses were summarized as percentages. Categorical data were analyzed using the chi-square test. Depending on the number and types of responses, regression analyses was performed to understand the association between various predictor variables and impact on domestic responsibilities using SPSS version 25. The study is registered with the Clinical Trials Registry of India—CTRI/2020/11/029174.

## RESULTS

### Demographics

The survey was disseminated among Indian physicians in various specialties mainly via e-mail and also using the messaging app WhatsApp. We received 1,041 responses in 2 weeks of October 2020 with 62% (643/1,041) respondents identified themselves as men. The response rate was difficult to capture as messages were shared widely among participants and their colleagues. Descriptions used for the survey included nuclear family defined as a couple (married or living together) with or without children and a joint family included a family with more than one generation of residents living in the same house, usually related by blood. Also, consultants were defined as physicians who had completed specialty training and were working independently (without supervision) and trainees as physicians pursuing a degree and working under the supervision of a consultant.

Demographic characteristics of the respondents are shown in Table 1. Most respondents belonged to the 31-40 years age group (men 253/643; 39.3%; women 146/393; 37.6%); 81% (318/393) of women respondents and 86% (554/643) of the men were consultants, and 45% (477/1,041) of the respondents belonged to surgical and allied specialties. The various specialties among the respondents were anesthesia, critical care, emergency medicine, general medicine, medical oncology, radiation oncology, surgery and allied fields (including obstetrics or gynecology, ear nose throat surgery, and plastic surgery), pediatrics, pathology, radiology, and others (to be specified).

**TABLE 1:**
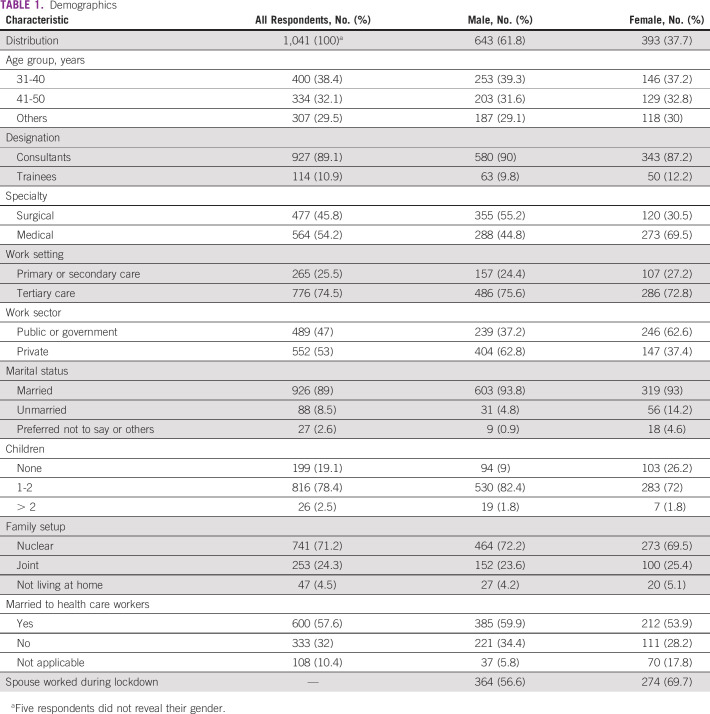
Demographics

**FIG 1:**
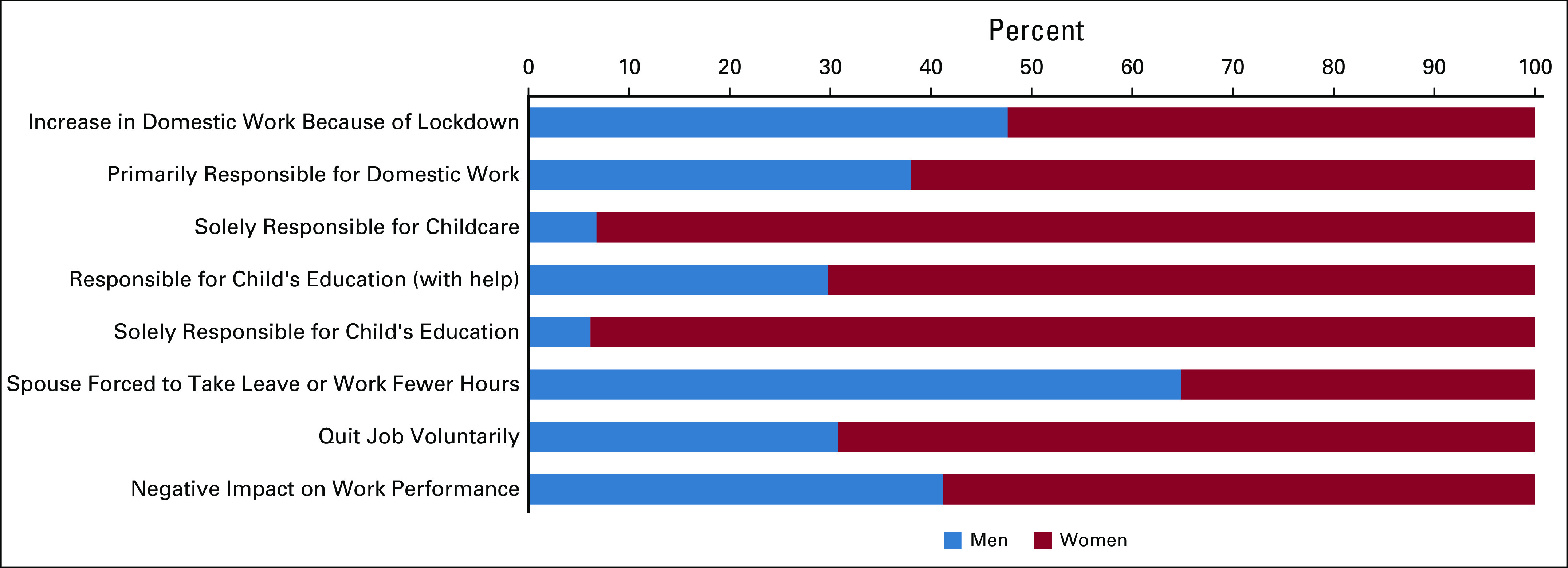
Impact of the pandemic on domestic chores, childcare, and work.

Majority of the respondents were married, 89% (926/1,041); 81% (840/1,041) had children and 24% (253/1,041) of the respondents lived in a joint family. Equal numbers of men and women respondents were married to health care workers, 63% (385/606) versus 65.6% (212/323), with 92.5% (297/321) of women's spouses working during the lockdown versus 67% (397/593) of men's spouses (*P* < .0001) . The impact of the lockdown on domestic responsibilities, childcare, and work is shown in Table 2 and Figure 1.

**TABLE 2:**
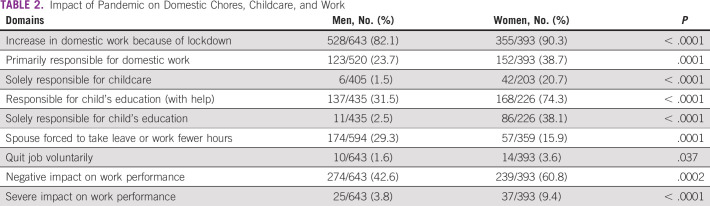
Impact of Pandemic on Domestic Chores, Childcare, and Work

### Impact on Domestic Responsibilities

Ninety percent of the women (compared with 82% men) felt that their domestic responsibilities had surged, with 129/393 of the women (32.8%) experiencing more than doubling of their responsibilities. Before the complete lockdown in April 2020, 83% (312/377) of the women and 78% (482/620) of the men relied on domestic help. However, during the period of restricted movement enforced by the government, from April to July 2020, 72% of the women respondents (268/373) and 60% of the men (356/588) did not have any domestic help.

During the lockdown, 81% of the women handled the domestic chores and groceries either alone or with some help versus 63% of the men. Also, 152/393 women versus 123/520 men (*P* = .00001) were solely responsible for the domestic chores.

### Impact on Childcare and Children's Education

Among the women, 290 out of 393 (73.7%) had children compared with 547 out of the 643 (85%) men (*P* < .0001); 58% (164/280) of the women and 61% (306/545) of the men had children younger than 12 years of age. Before the lockdown, men's spouses were primarily responsible for childcare in 112 out of 411 cases (25.9%) compared with 17 out of 225 women (7.5%). Majority of the women, 82% (167/203), were involved in childcare compared with 55% (222/405) of the men, but significantly more women (42/203) than men (6/405) were solely responsible.

Many parents (130/230—56% women and 184/231—79% men) felt that the children's educational burden had moderately increased with virtual schooling during the lockdown with approximately 83/461 (18%) finding this escalation to be extreme. Significantly higher proportion of women were directly responsible for their children's education than men (86 out of 226 women [38.1%] *v* 11/435 men [2.5%], *P* < .0001).

### Impact on Work

Majority of the physicians perceived that their work performance (clinical, teaching, administrative, and research, alone or in combination) was impacted by the increase in domestic chores, 61% women (239/393) compared with 274/643 (42.6%) men. Among men, 369/643 did not feel any impact of the increased domestic responsibilities on their work performance compared with 154/393 women (*P* < .00001). An extreme impact of household obligations was felt by 37/393 women compared with 25/643 men (*P* = .0002). Because of increased domestic responsibilities, fewer women's spouses (57/359) than men's (174/594, *P* = .00001) had to forcibly take leave or work reduced hours. About 3.5% (14/329) of the women respondents versus 1.5% (10/546) men voluntarily quit their jobs.

**FIG 2:**
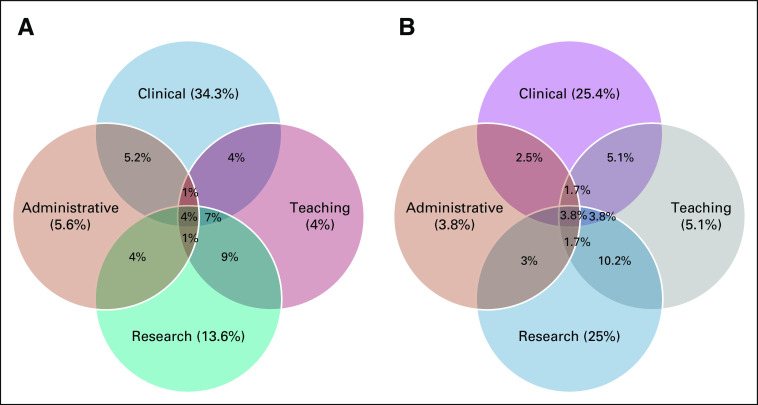
Impact on different aspects of work (clinical, research, teaching, and administration): (A) women and (B) men.

The impact on various aspects of work is demonstrated in Figure 2. A similar majority of both men, 514/643 (79.9%), and women, 320/393 (81.4%), felt they needed some flexibility or change in their working hours.

## DISCUSSION

Our survey of physicians in India during the pandemic indicates that the pandemic and the resultant lockdown caused considerable impact on physicians with regard to domestic responsibilities, childcare, and work. However, there seems to have been a disproportionately higher burden on women physicians compared with men. Whereas the increase in domestic chores were less inequitably shared between men and women, childcare responsibilities, especially related to helping with children's education, has been almost entirely been handled by women. There was also disproportionate adverse impact on women's careers compared with men physicians, both by their own perception as well as the need to take leave, work fewer hours, or even quit their jobs. We could not ascertain whether the respondents to our survey were representative. To the best of our knowledge, there are no published data on the marital status and/or family structure on physicians in India.

Gender inequity, whether apparent or not, has always existed in medicine but is being increasingly recognized and reported in recent years.^12,13^ Women physicians have had to make adjustments in their professional commitments to accommodate for managing their homes and bringing up their children.^14^ The traditional role of women as the primary caretakers at home has led to a constant struggle for women to maintain the delicate balance between professional aspirations and familial obligations. These compromises have had adverse impact on professional career progression for women in academic medicine. A recent study analyzing a database of more than 500,000 women medical graduates across 134 medical schools from the late 70s till recently^15^ found that there has been no narrowing of the gender gap when it came to promotion of women physicians to the rank of an associate or professor and this was evident across all branches of medicine, whether basic or clinical. The current COVID-19 pandemic threatens to widen this gender gap even further.^16^

The COVID-19 pandemic has created considerable difficulties for almost the entire global population. According to a recent McKinsey Survey, more than 1 million people dropped out of the workforce across the United States in the past 6 months, and 80% of them were women.^17^ The pandemic has been a particularly difficult time for physicians, who are under increased stress and higher risk for burnout by continuing to work extended hours, and in difficult circumstances; this has been even more problematic for women who continue to work more both at their workplace and home, trying to balance their professional and personal lives.^18^ A study published in *The Journal of the American Medical Association* evaluating the impact of domestic responsibilities on career satisfaction among physician-mothers^19^ found that mothers with more than five domestic tasks and in procedural specialties had higher career dissatisfaction.

This pandemic has impacted families of women physicians too; their spouses and other family members are also feeling the increment in domestic chores, needing to lend a helping hand.^20^ With more family members spending extended time at home, with limited avenues for recreation and restrictions on ordering food or eating out, there is a need for more sustained engagement with children, cooking and providing all meals. This has led to additional stress among family members in an already challenging time.^20^

Women (and men) often tend to underestimate women's contribution to the unrecognized and unpaid work required for managing a household, especially with young children.^21^ As the pandemic continues to rage, this work has intensified for women physicians, without a simultaneous and equivalent respite from professional duties. Domestic work that could earlier be delegated such as cooking, cleaning, groceries, laundry etc are now added to the list of household duties. Although men claim to be involved in domestic work and upbringing of the children, a New York Times poll suggested that men are prone to overestimation, with only 2% of the women agreeing to the men's claims of being solely responsible for providing care.^22^

For physicians with young children, there has been a complete lack of access to childcare facilities or employing childcare providers at home. With the unavailability of schools, play areas, and childcare, there was a need for constant childcare including getting acquainted to virtual education, which was challenging for both the children as well as parents.^23,24^ Most traditional Indian households have relied on grandparents to look after the grandchildren when both parents are working.^4^ However, the perceived threat and risk of infecting the high-risk and vulnerable elderly members in physicians' households may have led to enforcing social distancing even from grandparents. The lockdown has exacerbated the role of women physicians with young children in helping with schoolwork, with all the challenges of adapting to a new ecosystem of distant learning without the advantage of meeting their teachers and peers.^23,25^ Children's extracurricular activities, sports, and playtime also require considerable planning and with restrictions in movement and transportation, managing these tasks has become cumbersome.^20,25^

Despite a lot of negatives, the pandemic has also provided multiple avenues for virtual learning as well as teaching, for research, administration, and academics. Attempting to take advantage of these new opportunities while managing their familial roles is not feasible for most women physicians, with several publications showing decreased research output and published papers with women as first authors.^26-28^ A French study evaluating authorship of research articles among medical imaging journals found similar results.^29^

A similar survey on the impact of the pandemic was conducted recently in the United States with more than 2,700 respondents and the findings were presented at the Women in Medicine virtual summit.^30^ The study found that only 25% of the women physicians could continue to work without altering their schedules and less than half the emergency medicine physician-mothers felt adequately prepared for the pandemic personally or professionally, more so those with young children.^30^

The International Labour Organization defines unpaid work as any nonremunerated work carried out to sustain the well-being and maintenance of other individuals in a household or the community. ^31^ According to a database, Indian women spend an average of 351.9 minutes/day on unpaid work compared with 51.8 minutes/day by Indian men.^32^ In addition, these data also highlight the time poverty Indian women face—they spend 536.6 minutes/day on total paid and unpaid work compared with 442.3 minutes/day being spent by men. A recent survey performed to evaluate the impact of COVID-19 on the burden of unpaid work for women further underscores the gender disparities in domestic work in the Indian context.^33^ According to this survey, during the lockdown, a large proportion of married women witnessed a sharp increase in their unpaid work load; this trend was also seen prominently in employed women.

A telephonic survey in the Unites states found that 40% of institutions have no well-defined program to recruit, promote, or retain women in academic medicine.^34^ Efforts to make the working environment in medical fields more conducive for women requires efforts at individual, interpersonal, institutional, community, as well as policy levels.^35^ A systematic review evaluating mentorship programs designed for women in medicine found that these programs, regardless of the model, are met with high satisfaction and can help promote and retain women.^36^

Our study has several strengths and some limitations. To our knowledge, this is one of the largest surveys conducted on gender-based differences in domestic responsibilities and the impact of the pandemic and the resultant lockdown; it is also the only such survey conducted among Indian physicians. We had respondents from several specialties and is largely representative. The survey clearly highlighted that women physicians are primarily responsible for domestic chores, both before and after the pandemic or lockdown and are also more closely involved with their childcare and children's education. One of the limitations of our survey is that we were unable to calculate the response rate—this was because the survey was rapidly disseminated across chat groups and e-mailed to a database of e-mails gathered from previous conference attendees, estimating the denominator was difficult, if not impossible, and this may have led to a sampling bias. The entire survey was completed in 2 weeks, which makes the responses fairly homogeneous with regards to the timing of the questionnaire in relation to the pandemic. We had relatively fewer trainees who responded, and it is possible that the challenges they faced may not be adequately captured in the survey; this was difficult to control as we relied primarily on e-mail and chat message forwards to implement the survey. It is also possible that there may be some recall bias as the survey was conducted 2 months after the lifting of restrictions—this was done to ensure that all respondents had adequate time to readjust after the lockdown to reliably provide responses. Overall, our survey was fairly representative and generalizable across the physician community in India and probably other socioculturally similar countries.

In conclusion, we need to recognize that women's time and capacity to provide care is finite. Anxiety, stress, burnout, and depression are more common in women in general, and more so among women physicians. Inequitable distribution of domestic work adds substantially to their problems. Possible solutions include support systems for childcare, flexible working hours, extending leave to spouses or partners of women physicians, arrangements for outsourcing of domestic chores, and provision of psychosocial support, to name a few. Above all, recognizing that gender inequity is a real problem and acknowledging women's contributions to home and work should propel policy changes aimed at reducing these gaps. Unconventional solutions and support mechanisms are essential to maintain and nurture this important category of the medical workforce. The pandemic has demonstrated the possibilities of managing tasks that traditionally required physical presence such as academic conferences and administrative meetings virtually, along with new methods of schooling and bringing up our children. This flexibility should continue beyond the pandemic, not only to women but also to men, who could also contribute increasingly to domestic work. Organizations and hospitals that employ women physicians must work proactively to recognize and make provisions to reduce gender disparities in medicine across the world.
